# Bile acids as a “new hormone” in aging-related liver disease

**DOI:** 10.1093/lifemedi/lnag030

**Published:** 2026-08-07

**Authors:** Buyu Deng, Xingjian Li, Yue Li, Longchuan Han, Zhihui Zou, Yingting Zhang, Jing Luo, Xinxin Tang, Hui Kong, Chenxi Shi, Ying Huang, Hanzhi Luo, Yinkun Fu, Ming He

**Affiliations:** Institute for Translational Medicine on Cell Fate and Disease, Shanghai Ninth People’s Hospital, Key Laboratory of Cell Differentiation and Apoptosis of National Ministry of Education, Department of Pathophysiology, Shanghai Jiao Tong University School of Medicine, Shanghai 200025, China; Institute for Translational Medicine on Cell Fate and Disease, Shanghai Ninth People’s Hospital, Key Laboratory of Cell Differentiation and Apoptosis of National Ministry of Education, Department of Pathophysiology, Shanghai Jiao Tong University School of Medicine, Shanghai 200025, China; Institute for Translational Medicine on Cell Fate and Disease, Shanghai Ninth People’s Hospital, Key Laboratory of Cell Differentiation and Apoptosis of National Ministry of Education, Department of Pathophysiology, Shanghai Jiao Tong University School of Medicine, Shanghai 200025, China; Institute for Translational Medicine on Cell Fate and Disease, Shanghai Ninth People’s Hospital, Key Laboratory of Cell Differentiation and Apoptosis of National Ministry of Education, Department of Pathophysiology, Shanghai Jiao Tong University School of Medicine, Shanghai 200025, China; Institute for Translational Medicine on Cell Fate and Disease, Shanghai Ninth People’s Hospital, Key Laboratory of Cell Differentiation and Apoptosis of National Ministry of Education, Department of Pathophysiology, Shanghai Jiao Tong University School of Medicine, Shanghai 200025, China; Institute for Translational Medicine on Cell Fate and Disease, Shanghai Ninth People’s Hospital, Key Laboratory of Cell Differentiation and Apoptosis of National Ministry of Education, Department of Pathophysiology, Shanghai Jiao Tong University School of Medicine, Shanghai 200025, China; Institute for Translational Medicine on Cell Fate and Disease, Shanghai Ninth People’s Hospital, Key Laboratory of Cell Differentiation and Apoptosis of National Ministry of Education, Department of Pathophysiology, Shanghai Jiao Tong University School of Medicine, Shanghai 200025, China; Institute for Translational Medicine on Cell Fate and Disease, Shanghai Ninth People’s Hospital, Key Laboratory of Cell Differentiation and Apoptosis of National Ministry of Education, Department of Pathophysiology, Shanghai Jiao Tong University School of Medicine, Shanghai 200025, China; School of Exercise and Health, Shanghai University of Sport, Shanghai 200438, China; Institute for Translational Medicine on Cell Fate and Disease, Shanghai Ninth People’s Hospital, Key Laboratory of Cell Differentiation and Apoptosis of National Ministry of Education, Department of Pathophysiology, Shanghai Jiao Tong University School of Medicine, Shanghai 200025, China; Institute for Translational Medicine on Cell Fate and Disease, Shanghai Ninth People’s Hospital, Key Laboratory of Cell Differentiation and Apoptosis of National Ministry of Education, Department of Pathophysiology, Shanghai Jiao Tong University School of Medicine, Shanghai 200025, China; Cancer Epigenetics Institute, Fox Chase Cancer Center, Philadelphia, PA 19111, United States; Nuclear Dynamics and Cancer Program, Fox Chase Cancer Center, Philadelphia, PA 19111, United States; Institute for Cancer Research, Fox Chase Cancer Center, Philadelphia, PA 19111, United States; Institute for Translational Medicine on Cell Fate and Disease, Shanghai Ninth People’s Hospital, Key Laboratory of Cell Differentiation and Apoptosis of National Ministry of Education, Department of Pathophysiology, Shanghai Jiao Tong University School of Medicine, Shanghai 200025, China; Institute for Translational Medicine on Cell Fate and Disease, Shanghai Ninth People’s Hospital, Key Laboratory of Cell Differentiation and Apoptosis of National Ministry of Education, Department of Pathophysiology, Shanghai Jiao Tong University School of Medicine, Shanghai 200025, China; Department of Orthopaedics, Xinhua Hospital, Shanghai Jiao Tong University School of Medicine, Shanghai 200092, China

**Keywords:** bile acids, hormone-like signaling, farnesoid X receptor, G protein-coupled bile acid receptor 1, aging

## Abstract

Bile acids (BAs) have evolved from their classical definition as digestive surfactants to be recognized as potent endocrine signaling molecules that orchestrate systemic metabolism. Through activation of nuclear receptors, such as farnesoid X receptor (FXR), and membrane receptors, such as G protein-coupled BA receptor 1 (GPBAR1/TGR5), BAs regulate glucose, lipid, immune, and energy homeostasis. Aging reshapes BA synthesis, enterohepatic circulation, hydrophobicity, microbial biotransformation, and receptor responsiveness, thereby linking BA metabolism to hepatic senescence, inflammaging, and age-associated liver vulnerability. In turn, dysregulated BA signaling can amplify mitochondrial stress, endoplasmic reticulum stress, impaired autophagy, immune dysfunction, and senescence-associated secretory phenotypes, suggesting a bidirectional relationship between BA remodeling and aging progression. This review critically examines the role of BAs as hormone-like mediators within the aging liver microenvironment, summarizes major BA species and receptor preferences, integrates gut microbiota–BA crosstalk, and evaluates the therapeutic potential and caveats of targeting the BA–FXR–TGR5 axis in aging-related liver diseases, including metabolic dysfunction-associated steatotic liver disease (MASLD), alcohol-associated liver disease (ALD), autoimmune liver diseases (AILD), chronic hepatitis B (CHB), and hepatocellular carcinoma (HCC).

## Introduction

Bile acids (BAs) are synthesized in the liver from cholesterol and are crucial for the digestion and absorption of lipids in the small intestine. Although traditionally associated with lipid metabolism, recent studies have revealed that BAs also serve as important signaling molecules, regulating various metabolic processes. These signaling effects occur through receptors such as the farnesoid X receptor (FXR) and the G protein-coupled BA receptor 1 (GPBAR1, also known as TGR5). By influencing glucose metabolism, lipid regulation, energy homeostasis, and immune signaling, these receptors have become promising therapeutic targets for metabolic and liver diseases. We specifically place the BA axis within aging biology, because senescent hepatocytes, immune aging, mitochondrial–lysosomal stress, impaired proteostasis, and gut microbial remodeling can reshape both BA production and BA responsiveness. Recent studies on cellular senescence, T-cell aging, healthy-longevity microbiota, microbial BA modification, and global liver disease burden further support the need to analyze BA signaling as an age-sensitive liver–microbiome–immune network [1, 2]. We also examine the molecular mechanisms of BA signaling, their roles in synthesis and degradation pathways, and their increasing significance in aging-related liver diseases.

## BAs as a “new hormone”

Traditional studies suggest that BAs are metabolic substances in the body and mainly play a role in the metabolism of cholesterol [3]. Nowadays, however, increasing evidence supports BAs as endocrine-like metabolic signals, or “new hormones” [4], because they are secreted and recirculated after meals, bind nuclear and membrane receptors, and activate feedback loops that regulate their own synthesis, transport, and systemic metabolic output [4, 5]. Unlike classical hormones secreted from endocrine glands, BAs are metabolic intermediates whose signaling potency depends on chemical species, conjugation status, gut microbial conversion, intestinal reabsorption, and hepatic clearance. This distinction is important in aging, because each of these layers is remodeled during senescence, chronic liver disease, and microbiome dysbiosis [6].

To clarify this concept, we added a systematic comparison between classical hormones and BA-mediated hormone-like signaling (Table 1).

**Table 1.** Conceptual comparison between classical hormones and BA-mediated hormone-like signaling in aging.

Feature	Classical endocrine hormones	BAs as hormone-like signals	Aging implication
**Origin**	Endocrine glands/cells	Host hepatic cholesterol catabolism plus microbial modification	Aged liver and dysbiosis alter BA quantity and quality
**Release**	Pulsatile or stimulus-dependent blood release	Postprandial biliary secretion and enterohepatic cycling	Meal pattern, motility, gallbladder function, and bile flow change with age
**Circulation**	Systemic circulation to distant targets	Enterohepatic circulation with systemic spillover	Plasma BAs may indicate hepatic–intestinal perturbation
**Receptors**	Dedicated membrane/nuclear receptors	FXR, TGR5/GPBAR1, PXR, VDR, S1PR2, and other sensors	Aging changes receptor expression and inflammatory/metabolic output
**Feedback**	Classical endocrine axes	FXR–SHP–CYP7A1/CYP8B1 and intestinal FXR–FGF15/19–FGFR4	Feedback fragility may cause toxic accumulation or insufficient signaling
**Microbiome**	Usually indirect	Central for BSH, deconjugation, 7α-dehydroxylation, and epimerization	Dysbiosis shifts BA signaling toward inflammatory/cytotoxic profiles
**Therapy**	Replacement, antagonism, receptor modulation	FXR/TGR5 agents, sequestrants, hydrophilic BAs, microbiota remodeling	Frailty, polypharmacy, and cholestasis require individualized strategies

Abbreviations: BAs, bile acids; FXR, farnesoid X receptor; TGR5, Takeda G protein-coupled receptor 5; GPBAR1, G protein-coupled bile acid receptor 1; PXR, pregnane X receptor; VDR, vitamin D receptor; S1PR2, sphingosine-1-phosphate receptor 2; SHP, small heterodimer partner; CYP7A1, cholesterol 7α-hydroxylase; CYP8B1, sterol 12α-hydroxylase; FGF15/19, fibroblast growth factor 15/19; FGFR4, fibroblast growth factor receptor 4; BSH, bile salt hydrolase.

### Autoregulation of BA metabolism

It is known that there are multiple synthetic and regulated molecules in the anabolic process of BAs. Recent studies have revealed that BAs serve dual roles: they function as physiological detergents to aid in the absorption, transport, and distribution of fat-soluble vitamins and dietary fats, and they also act as signaling molecules that activate nuclear receptors, thereby regulating BA and cholesterol metabolism [7]. In other words, BAs have been found to influence triglycerides, cholesterol, energy, and glucose balance, while also controlling their own synthesis and enterohepatic circulation as feedback signals within their metabolic pathways. Acting as signaling molecules, BAs can trigger various signaling pathways, with their regulatory mechanisms receiving the most focus in scientific research.

BAs activate the FXR, a type of nuclear receptor, in the liver. FXR, first reported in 1995 [8], is a nuclear receptor activated by BAs, primarily expressed in the liver and intestine. Upon being activated by BAs, FXR forms a heterodimer with the retinoid X receptor (RXR), which then stimulates the expression of the small heterodimer partner (*SHP*) gene. This interaction is a key step in the regulation of BA metabolism, signaling pathways [9] and subsequently suppresses the nuclear receptor, thereby inhibiting the transcription of the rate-limiting enzymes cholesterol 7ɑ-hydroxylase (CYP7A1) [10] and sterol 12ɑ-hydroxylase (CYP8B1) [11] and liver receptor homolog1 (LRH-1). This pathway is a key regulatory pathway for BA biosynthesis. Moreover, FXRα-mediated *Fgf15* induction in the intestine was also reported as an auto-regulatory pathway of BAs [12].

Moreover, there is significant interest in a specific G protein-coupled receptor (GPCR) for BAs, known as TGR5. TGR5, as a member of the GPCR class rhodopsin subfamily (class A) [13], was first discovered by Maruyama et al. in 2002 [14], and TGR5 was recently identified as a membrane-bound receptor that is activated in response to BAs [14], providing new ideas for various aspects of BA homeostasis. At present, research has demonstrated that TGR5 is involved in regulating the physiological processes of various tissues, including immune cells, liver, gallbladder, small intestine, and muscle [15] (Fig. 1).

**Figure 1.**
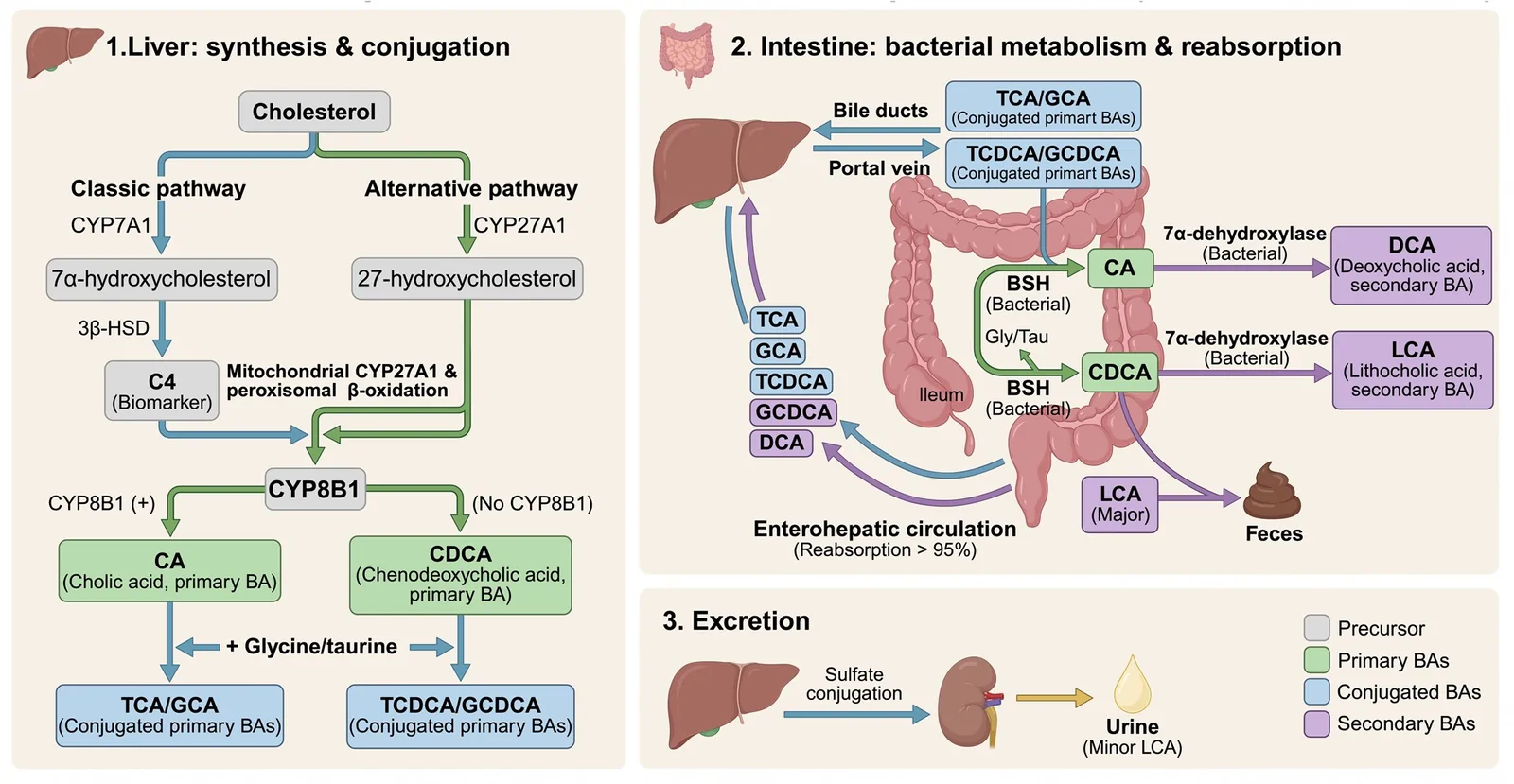
Metabolism of BAs. The classic pathway is initiated by cholesterol 7ɑ-hydroxylase (CYP7A1), while the alternative pathway is initiated by sterol 27-hydroxylase (CYP27A1). 3β-Hydroxysteroid dehydrogenase (3β-HSD) converts 7ɑ-hydroxycholesterol into 7ɑ-hydroxy-4-cholesten-3-one (C4); consequently, serum C4 levels have been established as a biomarker for assessing the rate of BA synthesis. Sterol 12ɑ-hydroxylase (CYP8B1) serves as the branching enzyme for cholic acid (CA) synthesis. In the absence of 12ɑ-hydroxylation, chenodeoxycholic acid (CDCA) is synthesized. Mitochondrial CYP27A1 catalyzes the oxidation of the steroid side chain, while peroxisomal β-oxidation cleaves the three-carbon unit to form the C24 cholanoic acid structure, which constitutes the backbone of most BAs. CA and CDCA represent the two primary BAs synthesized in the human liver. Upon synthesis, BAs are immediately conjugated with the amino acids taurine or glycine—forming species such as taurocholic acid (TCA) or taurochenodeoxycholic acid (TCDCA)—and are secreted into the bile. In the ileum, TCA and TCDCA undergo deconjugation catalyzed by bacterial bile salt hydrolase (BSH). Their 7ɑ-hydroxyl groups are subsequently removed by bacterial 7ɑ-dehydroxylase, resulting in the formation of deoxycholic acid (DCA) and lithocholic acid (LCA), respectively. These BAs (including TCA, TDCA, TCDCA, and the murine Tɑ-MCA and Tβ-MCA) are subsequently reabsorbed and recirculated back to the liver. LCA is primarily excreted via feces; however, a small fraction enters the liver, undergoes sulfation, and is ultimately excreted via urine. Created in BioRender. Boyu, D. (2026) BioRender.com/kqxy096.

A systematic summary of representative BA species, receptor preferences, aging‑associated relevance, and disease relevance is provided (Table 2).

**Table 2.** Representative BA species, receptor preferences, and aging-associated relevance.

BA species/group	Class/source	Main receptor preference	Aging-associated relevance	Disease relevance/caution
**CA**	Primary; CYP8B1-dependent	Weak FXR/TGR5	Age-related CA/CDCA shifts may alter hydrophobicity	Pool expansion; cholestatic/metabolic stress
**CDCA**	Primary; classic/alternative pathways	Potent endogenous FXR; moderate TGR5	Core FXR–FGF15/19–CYP7A1 feedback signal	Elevated conjugates reported in MASH/cholestasis
**DCA**	Secondary; microbial CA 7α-dehydroxylation	TGR5 agonist; context-dependent FXR	Dysbiosis may increase hydrophobic BA exposure	Pro-inflammatory; linked to injury and carcinogenesis
**LCA**	Secondary; mainly from CDCA	Potent TGR5; VDR/PXR; weak FXR	Longevity microbiota: context-dependent LCA signaling [16]	Adaptive at low levels but hepatotoxic when accumulated
**UDCA**	Hydrophilic tertiary/secondary BA	Weak FXR/TGR5; cytoprotective	May buffer hydrophobic BA toxicity during aging	First-line PBC therapy; incomplete response is common
**TCA/GCA**	Conjugated CA species	Species- and dose-dependent FXR/TGR5	Conjugation ratio varies with age, sex, diet, and microbiota	TCA can impair antiviral immunity and IFN response in CHB
**TCDCA/GCDCA**	Conjugated CDCA species	FXR-active primary conjugates	May reflect impaired hepatic BA handling in aged/metabolic liver	Often increased in MASLD/MASH cohorts
**TDCA/GDCA**	Conjugated DCA species	TGR5-active hydrophobic conjugates	May rise with microbial remodeling	Higher taurine/glycine ratios associated with HCC risk
**TUDCA**	Taurine-conjugated UDCA	Weak receptor agonist; chemical chaperone	Relevant to ER stress, proteostasis, and mitochondrial stress	Cytoprotective experimentally; clinical context matters
**TαMCA/TβMCA**	Murine primary BAs	TβMCA antagonizes intestinal FXR	Useful in rodent aging studies	Species-specific; minimal in humans
**isoLCA/3-oxoLCA/isoalloLCA**	Microbial LCA derivatives	Immunomodulatory; receptor selectivity evolving	Longevity-related microbial BA metabolism; validation needed [6]	Promising biomarkers; dose/species/toxicity require caution

Abbreviations: BA(s), bile acid(s); CA, cholic acid; CYP8B1, cytochrome P450 family 8 subfamily B member 1 (sterol 12α-hydroxylase); FXR, farnesoid X receptor; TGR5, Takeda G protein-coupled receptor 5, also known as GPBAR1 (G protein-coupled bile acid receptor 1); CDCA, chenodeoxycholic acid; FGF15/19, fibroblast growth factor 15/19; CYP7A1, cytochrome P450 family 7 subfamily A member 1 (cholesterol 7α-hydroxylase); MASH, metabolic dysfunction-associated steatohepatitis; DCA, deoxycholic acid; ER, endoplasmic reticulum; LCA, lithocholic acid; VDR, vitamin D receptor; PXR, pregnane X receptor; UDCA, ursodeoxycholic acid; PBC, primary biliary cholangitis; TCA, taurocholic acid; GCA, glycocholic acid; IFN, interferon; CHB, chronic hepatitis B; TCDCA, taurochenodeoxycholic acid; GCDCA, glycochenodeoxycholic acid; MASLD, metabolic dysfunction-associated steatotic liver disease; TDCA, taurodeoxycholic acid; GDCA, glycodeoxycholic acid; HCC, hepatocellular carcinoma; TUDCA, tauroursodeoxycholic acid; ER, endoplasmic reticulum; TαMCA, tauro-α-muricholic acid; TβMCA, tauro-β-muricholic acid; isoLCA, isolithocholic acid; 3-oxoLCA, 3-oxolithocholic acid; isoalloLCA, isoallolithocholic acid.

### Transduction and regulation effects of BAs on other metabolic pathways

Firstly, BAs function as signaling molecules involved in lipid metabolism. Francis et al. [17] reported that the nuclear receptors PPARα and LXRα regulate hepatic lipid metabolism, encompassing various stages of lipoprotein synthesis and breakdown. Specifically, these receptors influence high-density lipoprotein (HDL) metabolism by promoting the synthesis of Apolipoprotein A-I and Apolipoprotein A-II [18, 19], boosting the transfer of surface components from triglyceride-rich particles to HDL, and decreasing the replacement of HDL cholesterol with triglycerides [18]. Moreover, nuclear receptors also act on key hepatic and extra-hepatic steps of the synthetic and reverse cholesterol transport pathway. For example, Urizar’s studies [20] suggest that FXR receptors activated by BAs can regulate the expression of the *PLTP* gene *in vivo*, resulting in the production of proteins that facilitate the transport of cholesterol from tissues back to the liver for excretion via bile.

BAs and their receptors can play regulatory functions in glucose metabolism. Within the liver, BA–FXR signaling acts as a modulator of hepatic carbohydrate metabolism [21] that reduces hepatic gluconeogenesis and induces hepatic glycogen synthesis to regulate postprandial glucose levels [22]. FXR signaling in the intestine facilitates glucose 6-phosphate (G6P) absorption, though additional studies are required to fully determine its effects [23]. Xie et al. [24] suggested an alternative mechanism in which intestinal FXR signaling could influence the regulation of gluconeogenesis in the liver. In addition, TGR5, a key receptor for cell surface BAs, was shown to increase cAMP production and promote the secretion of glucagon-like peptide-1 (GLP-1) in the murine intestinal secretin tumor cell line (STC-1) [25], a process that may involve energy metabolism and intracellular calcium mobilization [26].

In addition, BAs act on the mechanical homeostasis of energy. TGR5 activation leads to increased energy expenditure in brown adipocytes and skeletal muscle by inducing the cAMP-dependent enzyme type 2 iodothyronine deiodinase, which activates thyroid hormone. The BA–TGR5–cAMP–D2 signaling pathway transforms inactive thyroxine (T4) into its active form, triiodothyronine (T3) [27], which directly affects the regulation of thyroid hormone for energy generation, and it plays a crucial role in regulating energy balance and can be targeted to enhance metabolic regulation. Sato et al. [28] reported a potential role of oleuropein and oleanolic acid as agonists of TGR5 in ameliorating metabolic disorders.

### Clinical significance of BAs as a therapeutic target

Given the significant role that BAs and their receptors have in the regulation of BAs, glucose, lipids, energy, and other lesser-known metabolic processes, they are increasingly recognized as a “novel hormone” with potential applications in clinical therapy. Studies have identified the role of BAs in obesity [29], metabolic dysfunction-associated steatotic liver disease (MASLD) [30, 31], diabetes mellitus [32], inflammatory bowel disease (IBD) [33], inflammation and cancer of the gastrointestinal tract [34], as well as neurodegenerative and neurological diseases [35]. Recent *Science China Life Sciences* (SCLS) studies also highlight cholesterol handling in the liver, hepatocellular glucose–lipid metabolism, microbiome-associated obesity, and liver disease burden as interlinked contexts in which BA signaling may be therapeutically relevant [36–38]. Other unproven common diseases may also be associated with them. Therefore, treating BA as a new signaling molecule and as a target is a new idea for various chronic diseases in the future, which may eventually be manifested as the improvement of aging [39]. Indeed, targeted natural and synthetic compounds focusing on BA-related signaling pathways have recently been developed, alongside research into mechanisms linked to aging. Nevertheless, BA signaling involves intricate pathways and multiple receptors, making the process of developing new therapeutics and treatment strategies lengthy and requiring extensive testing and refinement. In conclusion, BAs deserve more attention as “new hormones” or “new signaling molecules”.

### Receptors for BAs

#### TGR5

TGR5, part of the GPCR family characterized by seven transmembrane regions, is encoded by a single exon located on human chromosome 2q35 [14, 40]. TGR5 is distributed across multiple tissues and organs, exhibiting varying levels of expression. The highest expression is observed in the placenta and spleen, while moderate levels are found in the lungs, liver, stomach, small intestine, and adipose tissue. In contrast, lower expression levels are noted in the kidneys, skeletal muscle, and pancreas [41]. TGR5, functioning as a membrane receptor, can be internalized into the cytoplasm upon interaction with its agonist. This process triggers signal transduction via G_s_-protein-mediated cAMP accumulation, leading to the swift activation of downstream kinases [42] (Fig. 2). The agonists of TGR5 include, but are not limited to, BAs, INT-767 [42], SB-756050 [43], CA7S [44], and ZY12201 [45], in which the potency order of BAs to activate transfected Chinese hamster ovary (CHO) cells was taurolithocholic acid (TLCA) > LCA > DCA > CDCA > CA. TGR5 is also vital in regulating multiple key cellular signaling pathways, including the AKT–mTOR–liver inhibitory protein (LIP) axis [46], nuclear factor κB (NF-κB) [47], signal transducer and activator of transcription 3 (STAT3) [48], extracellular signal-regulated kinases (ERK) [49], and TGR5–β-arrestin signaling axis involving proto-oncogene tyrosine-protein kinase Src (SRC) [50]. It has been demonstrated that activating TGR5 through agonists can modulate a range of physiological functions, including glucose homeostasis [26], lipid metabolism [29], bile homeostasis [51], gallbladder relaxation [52], and BA-induced itch and analgesia [53].

**Figure 2.**
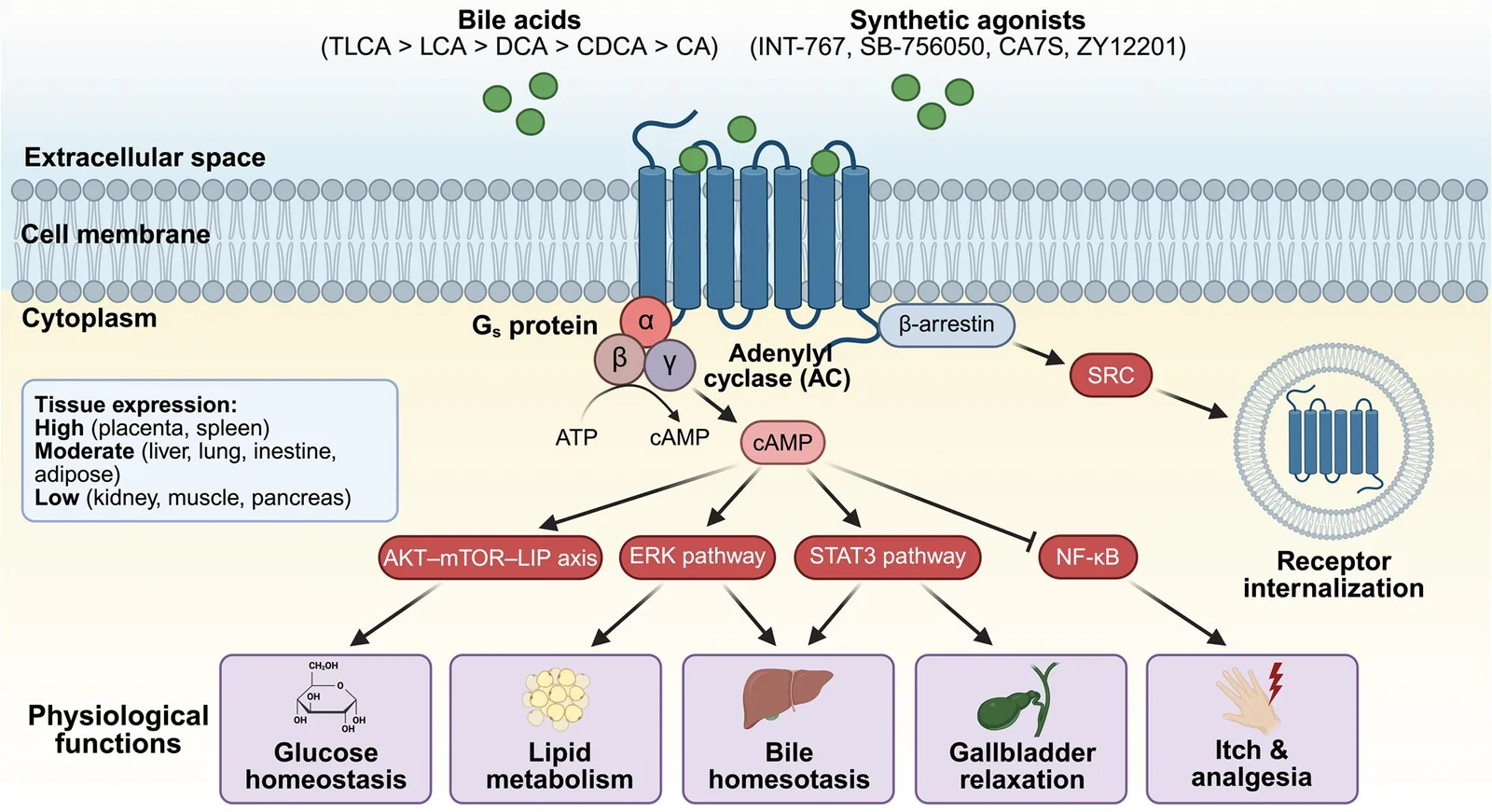
Schematic representation of the TGR5 signaling network and downstream physiological effects. Activation of the transmembrane GPCR TGR5 is initiated by ligand binding in the extracellular space, involving either endogenous BAs (with a potency rank order of TLCA > LCA > DCA > CDCA > CA) or various synthetic agonists (e.g., INT-767, SB-756050). This triggers a dual intracellular signaling cascade: the canonical Gs-protein-coupled pathway leading to adenylyl cyclase (AC)-mediated cAMP accumulation, and a non-canonical β-arrestin-dependent pathway that involves SRC activation and receptor internalization. The second messenger cAMP subsequently orchestrates multiple downstream kinase axes, including AKT–mTOR–LIP, ERK, and STAT3, while concurrently exerting inhibitory effects on NF-κB signaling. These diverse molecular pathways converge to regulate critical physiological functions, ranging from metabolic homeostasis (glucose, lipid, and bile regulation) to gallbladder motility and sensory responses such as itch and analgesia. The inset indicates the differential tissue expression pattern of TGR5. Created in BioRender. Boyu, D. (2026) BioRender.com/64at6gb.

From an aging perspective, the biological outcome of TGR5 activation is expected to depend on the BA species that dominate the aged BA pool. Hydrophobic secondary BAs such as LCA and DCA are potent TGR5 ligands, whereas changes in microbial deconjugation and 7α-dehydroxylation can alter their availability [6]. Therefore, TGR5 signaling should be interpreted together with age-related gut microbial remodeling, inflammaging, and tissue-specific receptor expression rather than as a static pathway.

#### Farnesoid X receptor

In 1995, the FXR was initially discovered as an orphan nuclear receptor activated by farnesol metabolites [8]. Shortly afterward, BAs were identified as the natural ligands for FXR [54]. FXR was later classified as NR1H4 (nuclear receptor subfamily 1 group H member 4). There exist two different *FXR* genes in mammals: *Fxrα* and *Fxrβ* [55]. In the human body, *FXRα* can encode four different isoforms (FXRα1–α4) by alternating promoters and N-terminal sequences (due to alternative splicing and promoter diversity) [56]. *FXRβ*, however, is classified as a pseudogene that cannot encode a functional protein in the human body.

All FXR isoforms, as members of the nuclear receptor (NR) superfamily, possess the characteristic modular domain structure. This includes a highly disordered N-terminal domain (NTD), a conserved DNA-binding domain (DBD), a hinge region, and a ligand-binding domain (LBD) [57]. The NTD contains a ligand-independent transcriptional activation region known as activator function-1 (AF-1), which facilitates transcriptional activation by coordinating coregulator proteins [9]. The DBD features two zinc finger motifs, each formed by four cysteine residues and a zinc ion, which fold inwards toward one another [58]. The carboxy-terminal end of the zinc fingers in DBD contains two helices (H1 and H2) [9]. These two helices are arranged perpendicularly, and, along with the zinc fingers, contribute to a more globular conformation of the DBD [58]. The two zinc finger domains differ in both structure and function. The first contains the DNA recognition helix, which interacts specifically with the major groove to form precise contacts with DNA bases. Conversely, the helix in the second zinc finger establishes less specific interactions with the DNA backbone [58]. The hinge region is a flexible and short linker positioned between the DBD and the LBD [57]. The LBD, an intricate signaling domain, has the ability to not only attach to ligands but also collaborate with co-regulator proteins directly. The overall conformation of LBD is composed of 12 α-helices (H1–H12) that fold into three parallel layers, creating an alpha-helical sandwich [59]. The LBD can be divided into three distinct regions: the ligand-binding pocket (LBP), the activation function-2 (AF-2) helix, and the dimerization interface. The LBP is composed of a group of hydrophobic residues that form a pocket, which accommodates its ligands, such as BAs. The hydrophobic nature of the pocket allows it to interact with the hydrophobic portions of the ligand molecule, while polar or charged groups of the ligand interact with specific residues lining the pocket [9, 60–62]. The AF-2 helix, consisting of helices 3, 4, and 12, interacts with co-activators to modulate the transcriptional activity of FXR. It has been confirmed that when binding to different ligands, H12 undergoes dynamic conformation changes, which causes the AF-2 to reorient and interact with various co-regulator proteins [62–64]. The dimerization interface is a region that allows two FXR molecules to bind together, which is necessary for the activity of FXR.

Alternative splicing and the use of distinct promoters lead to structural differences among the FXR isoforms. FXRα3 and FXRα4 feature an extended N-terminus. Moreover, FXRα1 and FXRα3 contain an insertion of four amino acids (MYTG) directly next to the hinge region [55, 65].

From an aging perspective, FXR signaling is neither static nor uniformly protective; its biological output depends on tissue compartment, ligand composition, and metabolic context. In senescence-accelerated mice, hepatic FXR expression and DNA-binding activity are reduced in parallel with steatosis and impaired fatty-acid oxidation, whereas diminished FXR activity contributes to the defective regenerative response of the aged liver; pharmacological activation of FXR improves regeneration through FOXM1B-dependent transcription [66, 67]. Aging also affects the intestinal arm of this pathway. One human–mouse aging study linked microbiota-dependent changes in BA metabolism to reduced ileal FXR–FGF19 (human) /FGR15 (mouse) signaling in older men and aged male mice, consistent with the established ability of microbial BA conversion to determine intestinal FXR tone [68, 69]. Given that the hepatic FXR–SHP–CYP7A1 pathway and the intestinal FXR–FGF15/19–FGFR4 pathway provide complementary feedback control over BA synthesis and enterohepatic flux [70, 71], disruption of either arm may reduce the capacity of the aging liver to adapt to metabolic and cholestatic stress. However, the consequences of manipulating FXR remain context-dependent: global FXR deficiency produces age-progressive steatohepatitis-like liver injury, whereas intestine-selective FXR inhibition or combined hepatic FXR/SHP deletion improves selected metabolic endpoints in mice [72–74]. Accordingly, FXR-directed interventions in older individuals should be interpreted—and ultimately designed—with attention to tissue specificity, BA composition, metabolic status, and disease stage.

#### Other types of nuclear receptors and GPCRs

BA receptors are primarily classified into nuclear receptors and GPCRs. In addition to FXR, BA-responsive nuclear receptors include PXR, VDR, and constitutive androstane receptor (CAR). In addition to TGR5, membrane-associated BA-responsive receptors include sphingosine-1-phosphate receptor 2 (S1PR2), formyl peptide receptors (FPRs), and other context-dependent sensors. These receptors differ in ligand specificity, cell type distribution, and biological outcome; therefore, their relevance to aging-related liver disease must be evaluated in a disease- and species-specific manner.

PXR, also referred to as NR1I2 (nuclear receptor subfamily 1 group I member 2) and SXR (steroid and xenobiotic receptor), is activated by steroid-like compounds, including pregnane 21-carbon steroids [75]. Like most nuclear receptors, PXR possesses a typical protein structure that includes an N-terminal non-ligand-dependent activation function 1 (AF-1), a highly conserved DBD, a less conserved hinge region, a C-terminal LBD, and an AF-2 [76, 77]. PXR is predominantly expressed in the liver and gut, where it regulates the transcription of hepatic genes involved in phase I and II drug metabolism, including enzymes like CYP3A, CYP2B, UDP-glucuronosyltransferases, and sulfotransferases, as well as drug transporters such as multidrug resistance proteins (MDRs) and multidrug resistance-associated proteins (MRPs) [78]. PXR is activated at relatively high concentrations by various hydrophobic compounds, both endogenous and exogenous, including steroids, BAs, pharmaceuticals, pesticides, phytochemicals, and industrial chemicals [79].

The vitamin D receptor (VDR), a member of the nuclear receptor superfamily, mediates the actions of the active hormonal form of vitamin D, 1,25-dihydroxyvitamin D3 [1,25(OH)2D3] [80]. Nearly all nucleated cells contain the vitamin D-VDR endocrine system. VDR has the ability to regulate a wide range of conditions affecting organs such as the bowel, kidneys, bones, skin, and heart. For instance, specific elimination of VDR in mammary epithelium significantly reduces mammary gland development during puberty [81].

Sphingosine-1-phosphate receptor 2 (S1PR2) is a GPCR that can be activated by conjugated BAs, particularly taurocholate, and couples to G_i_, G_q_, and G_12/13_-dependent pathways [82].Through ERK, AKT, and Rho-associated signaling, S1PR2 may influence hepatocyte proliferation, cholangiocyte biology, inflammation, and fibrosis. This receptor is therefore more relevant to BA signaling in mammalian liver disease than yeast Sip2/Snf1 regulatory proteins, which are not BA receptors and were removed from the receptor framework [83].

Formyl peptide receptor (FPR) is expressed on the surface of various cell types, including neutrophils, monocytes, hepatocytes, immature dendritic cells, astrocytes, microglial cells, and coronary artery walls. Formyl peptide receptor-like type-1 (FPRL-1) is present on numerous cell types, such as phagocytes, leukocytes, epithelial cells, T lymphocytes, neuroblastoma cells, astrocytoma cells, and microvascular endothelial cells [84]. These receptors have recently been recognized for their ability to bind and be activated by N-formyl peptides, which are produced by bacteria and can also be released by damaged mitochondria after tissue injury [85]. BAs have been found to activate three main MAPK signaling pathways: ERK, JNK, and p38 MAPK. However, the complete range of effects resulting from this activation remains to be fully understood [4].

## Changes in BA metabolism during aging

Aging remodels BA metabolism at multiple levels, including cholesterol availability, cytochrome P450 (CYP)-dependent BA synthesis, canalicular bile secretion, transporter expression, intestinal reabsorption, microbial biotransformation, and receptor feedback. Human and animal studies indicate that cholesterol saturation in bile can rise with age, driven by increased hepatic cholesterol secretion and/or reduced BA synthesis [86]. However, not all studies agree on whether total BA synthesis uniformly declines with aging, suggesting that age, sex, diet, metabolic status, and gut microbiota may determine the direction of BA changes (Fig. 3).

**Figure 3.**
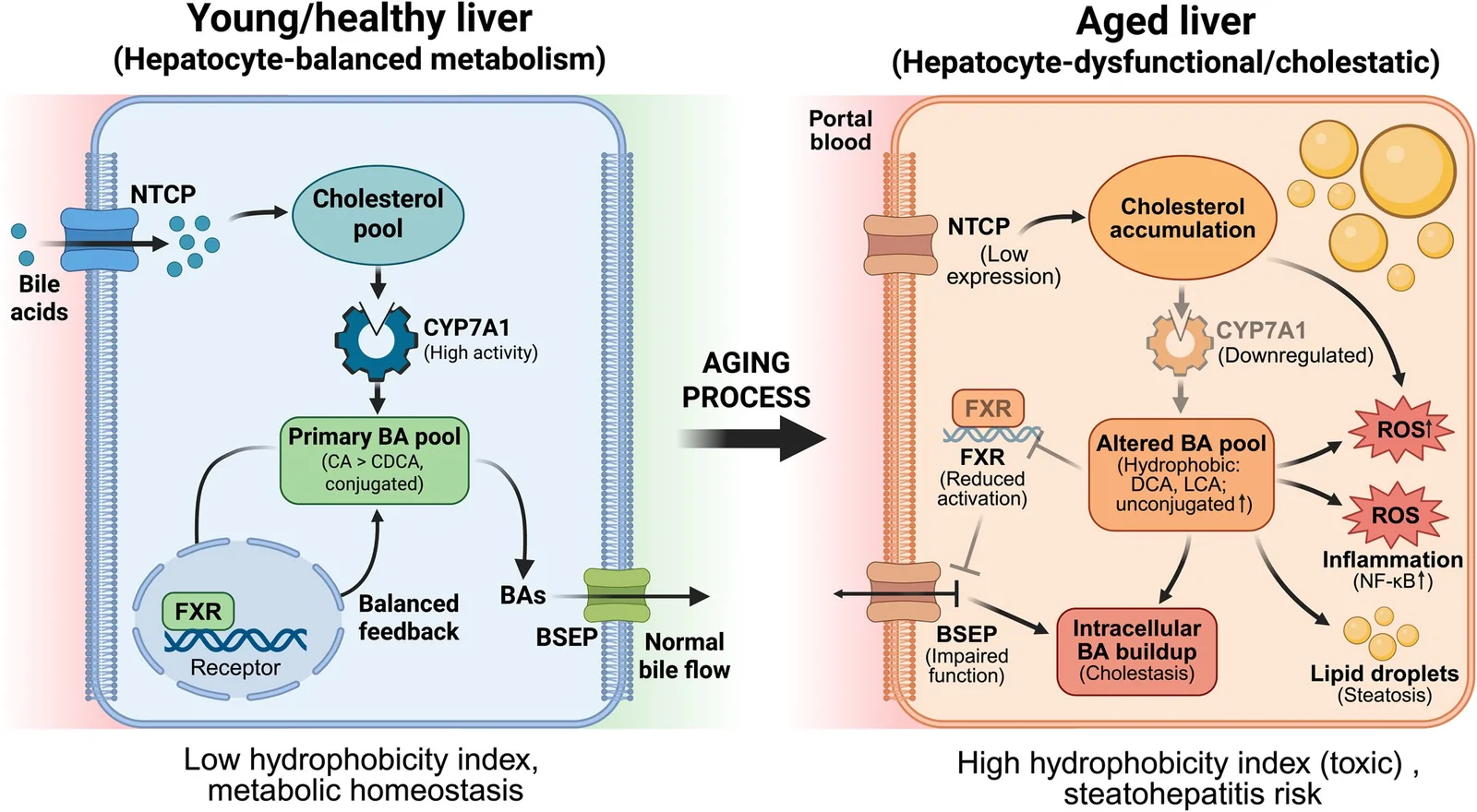
Changes in the BA content during aging. In young and healthy hepatocytes, NTCP-mediated BA uptake, CYP7A1-dependent conversion of cholesterol into primary BAs, FXR-mediated feedback regulation, and BSEP-mediated BA export are coordinately maintained. These processes support a predominantly conjugated primary BA pool, normal bile flow, a low BA hydrophobicity index, and metabolic homeostasis. In the aged liver, reduced NTCP expression, downregulated CYP7A1, decreased FXR activation, and impaired BSEP function are accompanied by hepatic cholesterol accumulation and a shift toward a more hydrophobic and unconjugated BA pool enriched in DCA and LCA. Impaired BA export promotes intracellular BA accumulation and cholestasis, whereas cholesterol accumulation and altered BA composition increase ROS production, NF-κB-associated inflammation, and lipid-droplet accumulation. Together, these changes contribute to steatosis and an increased risk of steatohepatitis. Created in BioRender. Boyu, D. (2026) BioRender.com/ias6mgp.

In previous experiments in mice [86, 87], cholesterol levels in both serum and liver increased with age, while hepatic phospholipid levels remained constant. The proportion of serum α-lipoprotein increased, whereas β-lipoprotein and pre-β-lipoprotein showed a slight decrease. Additionally, cholesterol and phospholipid levels in low-density lipoprotein (LDL), as well as cholesterol levels in high-density lipoprotein (LDL), rose with age. Bile flow, along with the secretion of cholesterol and BAs, decreased, whereas phospholipid secretion in bile increased [88]. HMG-CoA reductase enzyme activity, which regulates the biosynthesis of cholesterol, remained unchanged [89], while the level of cholesterol 7α-hydroxylation also decreased significantly with aging [90]. Bile secretion of α-deoxycholic acid and α-cholic acid decreased, but increased BA and deoxycholic acid resulted in increased CA/CDCA ratio. Levels of fecal lithocholic acid, β-cholic acid, and P10 (potentially ω-cholic acid) decreased with age, while other BA components either remained unchanged or showed a slight increase. In hypertensive rats (SHR), notable changes were observed earlier compared to Wistar-Kyoto (WKY) rats in several parameters, including cholesterol liver accumulation, serum lipoprotein percentage, liver enlargement, bile flow, bile secretion, increased BA pool, BA composition, and fecal BA excretion [91].

### Sex-specific ability of aging to alter BA metabolism

Aging changes the BA composition in the liver and serum in a sex-specific manner [92]. The serum of aged mice primarily contained conjugated primary BAs, including TαMCA (in males) and TαMCA + TβMCA (in females). Additionally, the ratio of conjugated to non-conjugated BAs, as well as primary to secondary BAs, changed in both liver and serum. The study also revealed that elderly female mice experienced more pronounced changes in liver BA composition, whereas elderly male mice showed greater alterations in intestinal BA composition.

Fu et al. [93] observed that in aging male mice, levels of UDCA, CA, and β-cholic acid gradually increased, while HDCA, CDCA, and DCA remained relatively constant. In female mice, TβMCA, DCA, CDCA, and UDCA levels gradually increased, whereas CA and HDCA showed stable levels. Correspondingly, female mice exhibited a decline in the expression of BA uptake transporters Ntcp and Oatp1b2, as well as the BA efflux transporter Bsep. In contrast, these transporter levels remained stable in aging male mice.

Similarly, according to the cross-sectional KarMeN study [94], age and sex were related to fasting plasma concentrations. Reduced BA synthesis with increasing age is one of the possible causes of elevated plasma cholesterol concentrations. Their study found that the overall decline in BA plasma concentrations with age occurred only in men, while most BA concentrations in women remained unaffected by aging.

### Anti-aging regulation of LCA and its isoforms

The intestinal flora and BAs of centenarians (individuals over 100 years old) contain various isoforms of LCA [95], which have antibacterial effects on Gram-positive multi-resistant bacteria, and can reduce lipid necrosis, improve mitochondrial structure, produce limited reactive oxygen species (ROS), and have a longer life span.

Beach et al. [96] discovered that LCA accumulates in yeast cell mitochondria, altering both the mitochondrial proteome and function. This enables mitochondria to act as signaling organelles that initiate an anti-aging transcription program involving transcription factors like Rtg1p, Rtg2p, Rtg3p, Sfp, Sfp1p, Yap1p, Msn2p, Msn4p, Skn7p, and Hog1p. Additionally, LCA induces age-related changes in protein levels both inside and outside of cells, impacting various cellular processes such as glycogen breakdown, glycolysis, stress response, and lipid metabolism.

### CYP7A1-related genes and the regulation of nuclear receptors

CYP7A1, belongs to cytochrome P450 (CYPs), is a phase I metabolic enzyme. Cytochrome P450 enzymes involved in BA homeostasis, corresponding nuclear receptors, and certain phase II genes are major contributors to the aging process. Age significantly influences P450 expression, with expression patterns showing lower levels of drug-metabolizing enzymes and related nuclear receptors in fetal and neonatal stages, increasing as the liver matures, and then declining rapidly during aging [97]. The expression patterns of FXR, CYP7A1, CYP8B1, CYP27A1, and their respective proteins were found to be similar. Protein expression levels corresponded closely with the changes observed in mRNA expression. The CYP7 family collaborates with CYP8 and CYP27 to regulate cholesterol and BA metabolism, ensuring metabolic homeostasis. In the elderly, disruptions in gut microbiota alter the activity of bile salt hydrolase (BSH)-producing bacteria, and reduced BSH activity leads to decreased BA metabolism and deconjugation [98]. This is because deconjugation of BAs occurs when BSH is over produced in the small intestine. During aging, the synthesis of BAs declines, along with reduced expression of the *CYP7A1* gene. This reduction is linked to alterations in the growth hormone/insulin-like growth factor (GH/IGF) axis [99]. IGF-I levels were positively correlated with serum levels of 7α-hydroxy-4-cholesterene-3-ketone (the precursor of BA), *CYP7A1* and *HNF-4* genes, while *HNF-4* and *CPF*/*LRH-1* genes were also correlated with *CYP7A1* gene expression. This indicates that IGF-I might have a role in regulating BA synthesis.

It is noteworthy that sex differences in BA concentration and composition in mouse serum and liver during aging may be attributed not only to the roles of BA transporters Ntcp and Oatp1b2 but also to sex-specific expression of CYP7A1 synthases [93].

Aging and FXR can act together to affect amino acid metabolism and the tricarboxylic acid cycle (TCA) cycle [100]. FXR was also found to be critical for age-related colonization of gut microbes. A high-fat diet decreases the urinary ratio of trimethylamine (TMA) to trimethylamine N-oxide (TMAO), thereby worsening hepatic steatosis through FXR inhibition. Additionally, FXR deficiency leads to metabolic deterioration, including elevated levels of 2-hydroxyvaleric acid and succinaemia linked to lactic acidosis. FXR knockout also results in reduced urine output, impaired BA synthesis, and altered cecal microbial community structure.

### Changes in BAs acting as molecular chaperones during aging

Some hydrophilic BAs, particularly UDCA and TUDCA, have been investigated as chemical chaperone-like molecules that alleviate endoplasmic reticulum stress and proteotoxic injury [101]. During aging, reductions in bile flow, impaired transporter function, and shifts toward hydrophobic BA species may weaken this cytoprotective component of the BA pool. This concept provides a mechanistic link between BA remodeling, loss of proteostasis, impaired autophagy, and cellular senescence in the aging liver [102, 103].

However, different conclusions have also been published. Galman et al. [104] suggested that there is no evidence that the synthesis of BAs decreases with age, and the age-dependent reduction in plasma LDL clearance is unlikely to be a result of reduced BA synthesis. The changes in BA metabolism in the aging process still need to be discovered and perfected.

### Causality between age-related BA alterations and aging

A key conceptual issue is whether BA remodeling is a cause or consequence of aging. Current evidence supports a bidirectional model. On one hand, aging alters hepatic cholesterol metabolism, CYP7A1/CYP8B1 expression, transporter function, bile flow, intestinal motility, and gut microbiota, all of which reshape the BA pool [86, 92, 94]. On the other hand, abnormal BA accumulation or loss of protective BA signaling can actively drive aging-related processes, including mitochondrial dysfunction, oxidative stress, endoplasmic reticulum stress, impaired autophagy, chronic inflammation, dysbiosis, and cellular senescence [36, 103, 105]. Thus, BAs should not be interpreted merely as passive biomarkers of aging liver injury.

Experimentally, the causal arm is supported by studies showing that hydrophobic BAs can induce inflammatory and proliferative signaling in hepatocytes and promote carcinogenesis, whereas selected interventions that modify BA signaling, intestinal FXR–FGF15/19 feedback, or microbial BA conversion can attenuate metabolic or alcohol-associated liver injury [106, 107]. Conversely, the consequence arm is evident because old age, sex hormones, metabolic dysfunction, and microbiota composition determine BA synthesis and composition. Future studies should therefore prioritize longitudinal human cohorts, age-stratified BA reference intervals, isotope-based BA flux measurements, and intervention trials that test whether normalizing BA composition can improve liver healthspan.

## Gut microbiota–BA crosstalk in aging and liver disease progression

### Microbial enzymatic remodeling of the BA pool

The gut microbiota is a major determinant of BA signaling because it converts host-derived primary BAs into a chemically diverse pool of secondary BAs. BSHs deconjugate taurine- and glycine-conjugated BAs, 7α-dehydroxylating bacteria convert CA and CDCA into DCA and LCA, and hydroxysteroid dehydrogenases generate oxo-, iso-, allo-, and other epimerized BA derivatives. These reactions alter BA hydrophobicity, receptor selectivity, antimicrobial activity, and enterohepatic recycling [6]. Because aging is accompanied by dysbiosis and altered intestinal barrier function, microbial BA metabolism provides a plausible mechanistic bridge between chronological age and liver disease susceptibility [2, 16].

### Aging-associated dysbiosis as a disease amplifier

In aging, the microbiota–BA axis can amplify liver injury through several converging mechanisms. First, reduced microbial diversity or altered BSH/7α-dehydroxylase activity can shift the BA pool toward conjugated primary BAs or hydrophobic secondary BAs, changing FXR and TGR5 tone. Second, age-related intestinal barrier dysfunction increases translocation of microbial products, coupling BA dysregulation to hepatic Toll-like receptor activation, Kupffer cell inflammation, and inflammaging; experimental evidence that *Christensenella tenuis* alleviates endotoxemia by inhibiting intestinal lipopolysaccharide translocation illustrates the importance of barrier control in metabolic disease [6].

### Therapeutic implications of microbiota-targeted BA remodeling

Therapeutically, the microbiota–BA axis suggests that BA receptor agonism alone may be insufficient in older patients. Potential strategies include probiotics or prebiotics that restore BSH-producing communities, antibiotics or bacteriophages that reduce harmful 7α-dehydroxylating activity, fecal microbiota transplantation in selected contexts, BA sequestrants to reduce hydrophobic BA exposure, and hydrophilic BAs such as UDCA/TUDCA to counter cytotoxicity. However, because the same BA species can be protective at physiological concentrations and toxic when accumulated, interventions should be guided by age-, sex-, disease-, and microbiome-stratified BA profiling rather than by total BA concentration alone [108–110].

## The role of BA metabolism in aging-related liver diseases

The burden and clinical severity of many chronic liver diseases rise with age, and advanced fibrosis, decompensation, and liver cancer are more frequently encountered in older populations. Aging is not merely a chronological background variable; it brings hepatic senescence, inflammaging, mitochondrial–lysosomal stress, reduced regenerative capacity, immune remodeling, gut barrier dysfunction, microbiota dysbiosis, and altered BA synthesis/transport. Therefore, the following disease sections emphasize how BA metabolism participates in liver aging by examining chronic hepatitis B (CHB), AILDs, alcohol-associated liver disease (ALD), MASLD/MASH, and hepatocellular carcinoma (HCC) under the aging background.

### Chronic hepatitis B

CHB represents a major global health concern and is the most prevalent form of viral hepatitis, with a significant risk of advancing to severe liver disease. The hepatitis B virus (HBV) genome consists of partially double-stranded relaxed circular DNA, which is regulated by the transcriptional activation of four key promoters (core, X, pre-S1, and pre-S2/S) and two enhancers (EnhI and EnhII) [111]. Cellular transcription factors recruited to binding sites in the HBV genome can regulate viral transcription. These include HNF4α, PPARα/RXRα, and FXR, among others. Most transcription factors bound to HBV naturally function to coordinate and regulate liver metabolism: HNF4α is involved in glucose metabolism, PPARα oversees fatty acid β-oxidation, and FXR is activated by BAs [112].

Numerous pieces of evidence have indicated a link between HBV infection and BAs. One study showed a significant increase in glycocholate 63-glucuronide, taurocholate, TCA, 31-sulfate ethanol cholate, and glycoside deoxycholate (GUDCA) in patients with hepatitis B-induced cirrhosis, highlighting their potential as biomarkers [113]. Moreover, the total BA to cholesterol ratio (TBA/TC) was found to be significantly elevated in patients with HBV-induced cirrhosis. Evaluating the TBA/TC ratio can serve as an additional marker for identifying significant liver fibrosis and cirrhosis in non-cholestatic chronic HBV infection [114]. Another study also demonstrated that serum total BAs (TSBA) can be used to predict hepatitis Be antigen (HBeAg), negative, borderline alanine aminotransferase (ALT), and high HBV DNA for CHB antiviral therapy [115]. Under the aging background, these BA-related indices should be interpreted together with fibrosis stage, immune aging, and comorbidity burden; recent SCLS work further supports extracellular matrix protein 1 (ECM1) as a promising serum biomarker for staging and monitoring fibrosis in CHB [116].

In many studies, the sodium taurocholate cotransporting polypeptide (NTCP), a hepatic BA transporter, has garnered significant attention. NTCP is a transmembrane transporter located on the basolateral membrane of hepatocytes, comprising nine transmembrane domains that oligomerize within the phospholipid bilayer. It utilizes a sodium gradient to transport two sodium ions along with a taurocholate molecule across the membrane. NTCP has two primary functions: the uptake of most sodium-dependent bile salts by hepatocytes, and its role as a cellular receptor for HBV, interacting with the pre-S1 domain of the HBV large envelope protein during viral entry. Specifically, the myristoylated preS1 (myr-preS1) peptide domain derived from the HBV L protein surface protein is linked to the NTCP to mediate viral entry (Fig. 4). Therefore, HepG2 cells reconstituted with HBV into NTCP are widely used as a convenient *in vitro* cell culture infection model for HBV replication studies, and a subclone that has isolated HepG2-NTCP-A3, as an improved *in vitro* HBV infection model, has a stable high infection rate [117]. König et al. [118] demonstrated that the binding of HBV to NTCP disrupts NTCP’s physiological function of BA transport, and this effect is specific to the liver (not for other proteins in the SLC10 carrier series to which NTCP is affiliated). This interference may stem from the NTCP molecular decision clusters that mediate HBV entry into the cell overlapping with those of BA transport [119]. What’s more, HBV often leads to bile stasis, so the search for mutations or therapies that inhibit viral infection but preserve BA transport is particularly critical. Zakrzewicz et al. [120] identified a stretch of the 139YIYSRGIY146 motif of NTCP in which certain tyrosine-specific mutations retain the protein transport function of NTCP while greatly reducing the probability of viral infection to achieve gene target therapy of CHB. Furthermore, studies have indicated that a reduction in bile salts triggers compensatory BA synthesis to maintain homeostasis. This effect is evidenced by increased activity of CYP7A1, the rate-limiting enzyme for cholesterol conversion to BAs, as well as reduced nuclear translocation of FXR and decreased expression of SHP, a core repressor of CYP7A1 transcription [121]. However, this compensatory mechanism is unable to fully stabilize BA levels, and experimental evidence suggests that FXR and SHP play only a limited role in regulating HBV biosynthesis [122]. Recently, the study of Ito et al. [123] identified INT-767, a BA derivative, which exhibits a potent inhibitory effect on HBV. This inhibition occurs through the activation of both FXR and TGR5 receptors, stimulating downstream signaling, and directly interacting with HBV particles instead of binding to NTCP. In summary, NTCP and BA transport disorders playing important roles in the pathogenic mechanisms of HBV have become novel targets for drug therapy.

**Figure 4.**
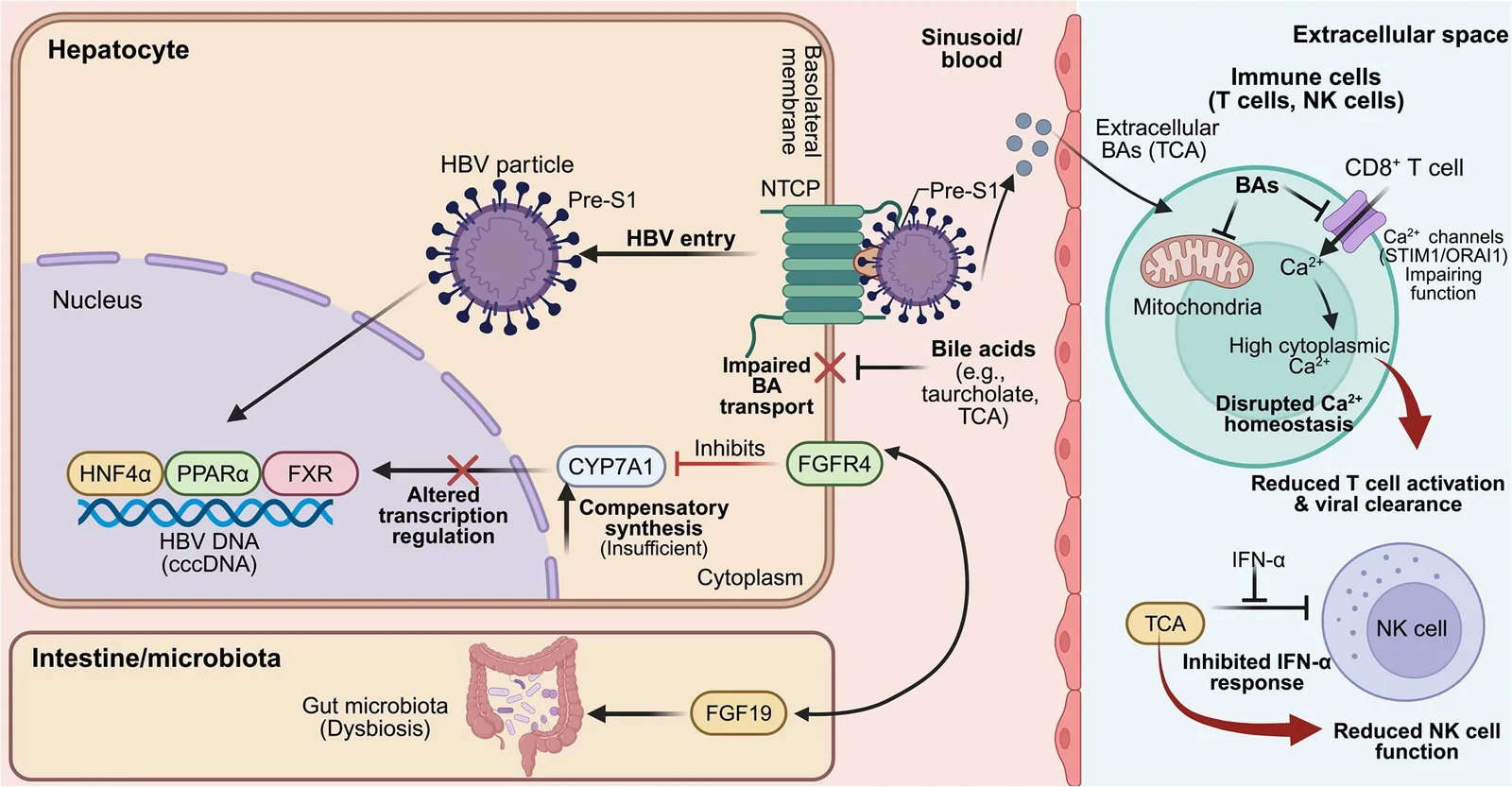
The role of BAs in the development of hepatitis B. The myr-preS1 peptide domain derived from the HBV L protein surface protein is linked to the NTCP to mediate viral entry and then combine with HepG2 cells, which is used as an infection model with stable high infection rate. Created in BioRender. Boyu, D. (2026) BioRender.com/jgj2d6a.

In addition, increased BA levels otherwise affect antiviral immunity and hinder HBV clearance. Research has demonstrated that BAs are crucial in mediating resistance to HBV infection by regulating T cell activation and metabolism. In a transgenic mouse model of HBV infection, decreased CD4^+^ and CD8^+^ T cell activation resulted in inefficient viral clearance. The underlying mechanism may involve BAs disrupting intracellular calcium homeostasis by inhibiting mitochondrial calcium uptake while increasing cytoplasmic calcium levels, thereby uncoupling and impairing the function of STIM1 and ORAI1 [124]. Moreover, similar mechanisms may also act on the treatment of CHB. Xun et al. [125] found that serum TCA inhibited the response to pegylated interferon-α (PegIFNα) in HBeAg-positive CHB patients. IFN-α is the standard treatment for CHB patients, working by inducing antiviral protein production via the JAK–STAT pathway and suppressing viral replication through immunomodulatory effects. TCA was found to reduce CD8^+^ T and NK cell activation and function both *in vitro* and *in vivo*, and it inhibited the immunomodulatory activity of IFN-α *in vitro*. Specifically, BA can change membrane fluidity and permeability (Ca^2+^) by embedding cell membrane, activate protein kinase C signaling pathway. BA suppresses the interaction of cytokine receptor ligands and intracellular pathway of cell activation. TCA activates TGR5, upregulates PD12 expression by inhibiting the production of IL-1, induces the downregulation of transforming growth factor β, and activates the expression of NK cell receptor NKG2D. Overall, the impact of BAs on the immune system in HBV infection may influence both disease progression and treatment outcomes.

Notably, the gut microbiota also plays a role in altering the BA pool in CHB due to the liver-BA–microbiota axis. Elevated FGFR4 bound to FGF19 in the intestine, downregulating CYP7A1 in hepatocytes and further reducing the synthesis of primary BA in CHB patients [126]. Similarly, FGF-19 was not sufficient to counteract the increase in serum BAs caused by cholestasis.

### Autoimmune liver diseases

Autoimmune liver diseases (AILDs) refer to a group of hepatic immune disorders of unknown origin, encompassing several chronic liver conditions such as primary biliary cirrhosis (PBC), primary sclerosing cholangitis, and autoimmune hepatitis (AIH). Primary biliary cholangitis and autoimmune hepatitis, in particular, have garnered significant attention due to their characteristic clinical features, including the presence of specific autoantibodies in serological tests [127]. The first-line treatment for autoimmune hepatitis involves immunosuppression, while primary biliary cholangitis is primarily treated with UDCA. Both conditions have the potential to progress to liver cirrhosis.

BA metabolism is suggestive for the diagnosis of AILDs. Ma et al. [128] identified BA levels as potential biomarkers for the early diagnosis and differentiation of the severity of primary biliary cholangitis (PBC) and autoimmune hepatitis (AIH). Specifically, LCA disrupts phospholipid and sphingolipid homeostasis via the transforming growth factor β signaling pathway, leading to elevated levels of CDCA, LCA, TLCA, and LCA + TLCA in PBC patients compared to those with AIH. Additionally, high BA levels may induce PBC, activate *FXR* gene transcription, and contribute to PBC development and LCA toxicity in patients [129]. Given that CDCA is converted to LCA by gut flora and then to TLCA by intestinal bacteria, it has been hypothesized that dysfunction of the gut microbiota may elevate the risk of AILDs, including PBC [130].

Further research indicates that BA metabolism is crucial in the progression of AILDs. Microbial metabolites, such as short-chain fatty acids (SCFAs) and BA derivatives, have been found to influence AILDs-related innate and adaptive immune responses, as well as inflammation [131]. Regulation of the enterohepatic circulation of BAs and BA-related signaling pathways may contribute to the pathogenesis of PBC. Relevant mechanisms include the protective effects of FXR activation in *Abcb4*-deficient cholangitis, FGF19-mediated regulation of BA homeostasis, the immunomodulatory effects of TGR5 activation, and disruption of the biliary bicarbonate “umbrella.” In the case of cholestasis, there may be interactions and synergies between several regulatory pathways (e.g., FXR and TGR5) [132]. In addition, the pregnane X receptor (PXR) and the G protein-coupled receptor 35 (GPR35) may also be involved in the regulation (Fig. 5). With regard to AIH, the current study shows that FXR protects mice from the development of ConA-induced liver injury by significantly lowering ALT and AST levels [133]. Recent work on PXR–p53-dependent hepatocyte protection and glutathione-CAR-dependent hepatic injury further suggests that BA-responsive xenobiotic receptors should be considered in aged or cholestatic immune-mediated liver injury [134].

**Figure 5.**
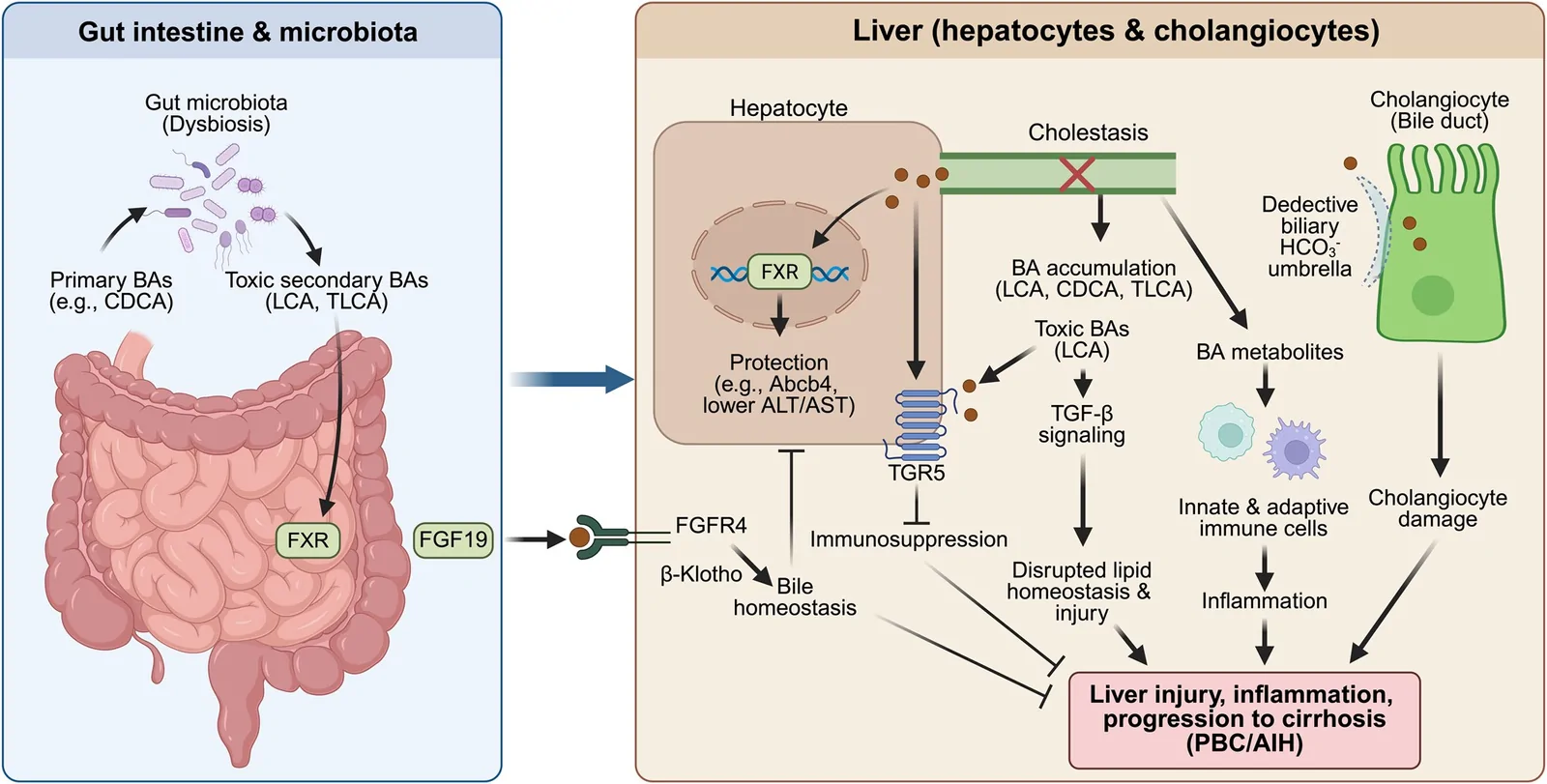
The role of BAs in the development of autoimmune liver disease. BA levels could potentially be used as biomarkers for the early diagnosis and differentiation of primary biliary cholangitis (PBC) and autoimmune hepatitis (AIH) severity. Elevated BA levels may induce PBC and *FXR* gene transcription in patients, which could contribute to the development of PBC and LCA toxicity. Through the transforming growth factor β signaling pathway, LCA elevates the levels of CDCA, LCA, TLCA, and LCA + TLCA in PBC patients compared to AIH patients. The regulation of BAs is related to human PBC in many pathways: (1) the important role of FXR activation in improving the development of Abcb4 cholangitis; (2) the regulatory function of FGF19 in bile homeostasis; (3) the immunosuppressive role of TGR5 receptor activation; (4) the defect of the bile bicarbonate “umbrella”; (5) PXR, GPR35 and other receptors. Different regulatory pathways could have interactions and synergies if cholestasis occurs. Created in BioRender. Boyu, D. (2026) BioRender.com/ie8glnv.

PBC and AIH are always concurrent in related studies and a considerable proportion of mixed diseases exists in the clinic, so autoimmune hepatitis of these cholestatic phenotypes is designated as “overlapping syndrome”, diagnosed by the “Paris criteria” [135]. This sets up new targets for the research on BAs.

### Alcoholic liver disease

ALD is a condition resulting from excessive alcohol consumption and includes hepatic steatosis, steatohepatitis, fibrosis, cirrhosis, and HCC. ALD is linked to disturbances in BA synthesis and enterohepatic circulation. Alcohol intake increases oxidative stress, disrupts lipid metabolism, damages the intestinal barrier, reshapes the gut microbiota, and causes cholestasis. In older adults, these insults intersect with reduced hepatic reserve, mitochondrial–lysosomal stress, immune aging, sarcopenia, and polypharmacy, making BA accumulation and dysregulated FXR/TGR5 signaling clinically more consequential BA [36, 136].

Research has demonstrated that FoxO3a transcription factors are crucial in the progression of alcoholic liver disease. Mechanistically, acute alcohol exposure reduces AKT-mediated phosphorylation of FoxO3a at the serine 253 site, leading to its accumulation in the nucleus of mouse liver cells. Notably, resveratrol activates SIRT1, which promotes deacetylation of FoxO3a, enhancing the transcription of the *Atg* gene in response to alcohol exposure. Both BAs and FoxO3a are involved in regulating hepatic autophagy. Studies indicate that BAs induce autophagosome accumulation in hepatocytes and esophageal cells, as well as increase LC3-II protein expression. Additionally, BAs hinder the completion of autophagy in hepatocytes by inhibiting the fusion of autophagosomes with lysosomes [137] (Fig. 6).

**Figure 6.**
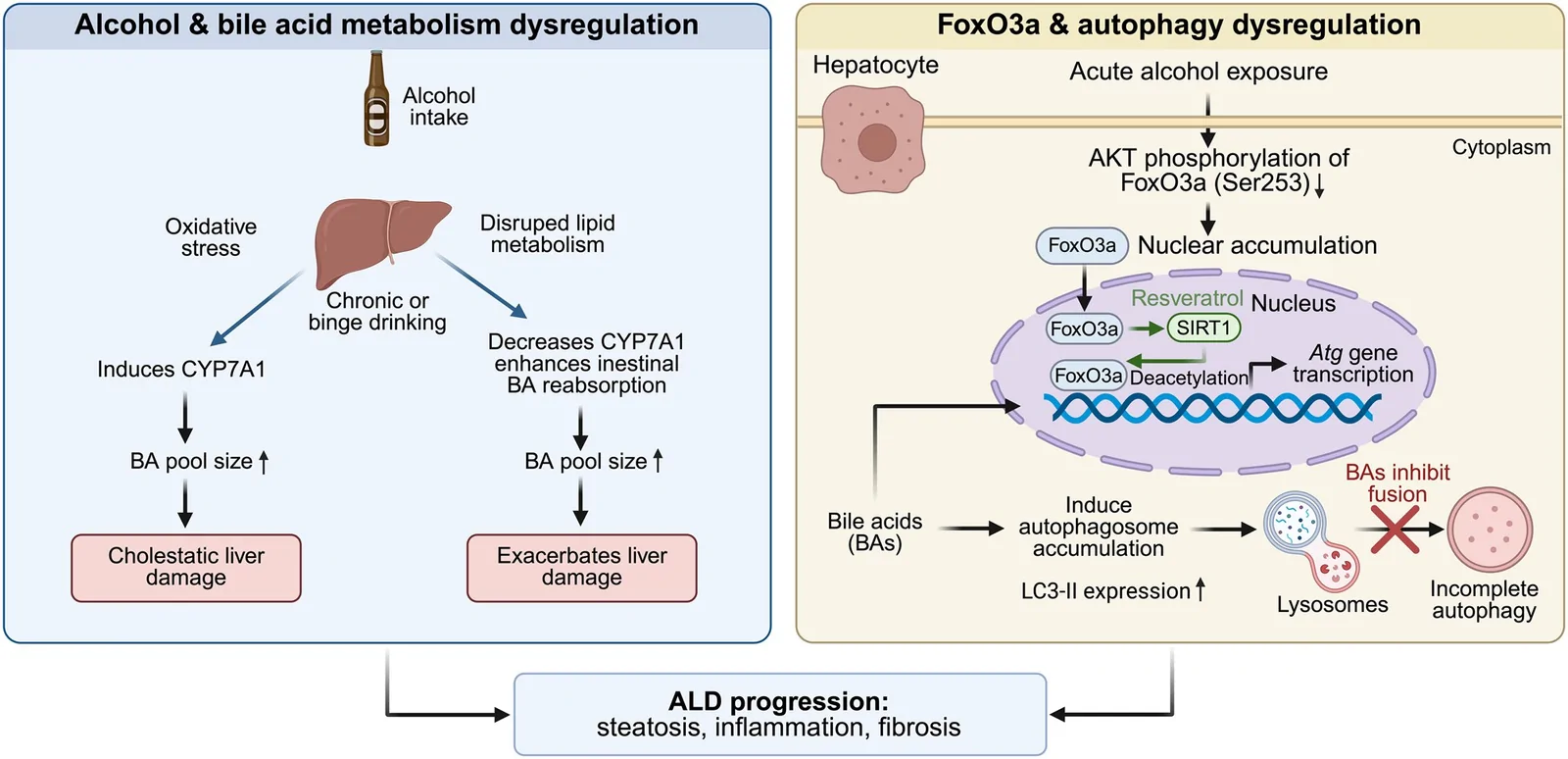
The role of BAs in the development of alcoholic liver disease. FoxO3a transcription factors are pivotal in the pathogenesis of alcoholic liver disease. In terms of mechanism, acute alcohol administration decreased FoxO3a’s serine 253 site phosphorylation by AKT, which led to an increase of FoxO3a in the mouse liver’s nucleus. Resveratrol, on the other hand, enhances FoxO3a deacetylation by activating SIRT1, thereby boosting the transcription of *Atg* in response to alcohol. Additionally, BAs and FoxO3a are crucial in modulating hepatic autophagy. Evidence suggests that BAs stimulate the buildup of autophagosomes in both hepatocytes and esophageal cells and elevate LC3-II protein levels. Conversely, BAs impede the completion of autophagy in hepatocytes by reducing the fusion of autophagosomes with lysosomes. Created in BioRender. Boyu, D. (2026) BioRender.com/i1j8zmt.

Some studies have found that chronic or binge drinking induces the expression of CYP7A1 and increases the size of the BA pool, leading to cholestatic liver damage. However, chronic binge drinking decreases CYP7A1 expression but enlarges the BA pool by enhancing intestinal BA reabsorption, which, in turn, exacerbates liver damage [138].

Another study found that long-term alcohol consumption led to overexpression in the genomic DNA of gut bacteria encoding choloylglycine hydrolase, which decomposes to BA in the gut. After alcohol intake, the content of unconjugated cholic acid in the small intestine increases. Decreased FXR activity in intestinal cells, resulting in increased expression of hepatic cytochrome P450 enzyme CYP7A1 protein and circulating cholic acid levels. A decrease in commensal gut flora can reduce intrahepatic CYP7A1 expression and lower the incidence of alcoholic liver disease, indicating that increased BA synthesis is dependent on gut microbiota [139]. Host–microbiome interactions that regulate hepatic glutathione and CAR-dependent pathways may therefore be relevant for mitigating oxidative liver injury in aged ALD [134].

Way et al. [140] found that BAs play an important role in cell proliferation and regulate cell proliferation by coupling to different G proteins. Specifically, TGR5 inhibits cell proliferation when coupled to G_i_, and promotes cell proliferation when coupled to G_s_. In some cell lines, TGR5 couples to both G_q_ and G_i3_, but only G_q_ exhibits signaling upon ligand binding. S1PR2 is coupled with G_i_, G_q_, and G_12/13_ proteins. G_i_ activates the phospholipase C (PLC)/IP3/DAG pathway, PI3K–Akt signaling, and the MAPK pathway, while G_q_ exclusively activates the PLC/IP3/DAG pathway. G_12/13_, on the other hand, triggers the Rho/ROCK, NF-κB, and PTEN pathways, leading to inflammation and stress fiber formation. BA activation is primarily mediated by binding to conjugated BAs and coupling with G_i_.

Zhang et al. [141] found that alcohol intake activates cannabinoid receptor type 1 and CREBH in the liver, thereby increasing the expression of CYP7A1 and CYP27A1, which in turn increases BA synthesis and BA pool accumulation. This leads to oxidative stress in the liver, cell proliferation, and an increase in liver weight. Studies have also found that alcohol intake alters the expression of BA transporters, including upregulating BSEP and MRP2 in the liver and ASBT and OSTβ in the liver and ileum, thereby increasing BA reabsorption. Previous experiments have demonstrated that altering the gut microbiota and its metabolic capacity can alleviate ALD by modifying the enterohepatic circulation of BAs.

### Metabolic dysfunction-associated steatotic liver disease

Metabolic dysfunction-associated steatotic liver disease (MASLD), once known as nonalcoholic fatty liver disease (NAFLD), is a leading global cause of liver disorders and is projected to become the primary contributor to end-stage liver disease in the coming decades. This condition affects individuals of all ages, including both adults and children [142]. MASLD encompasses a spectrum of conditions, ranging from simple fatty liver (NAFL) to metabolic dysfunction-associated steatohepatitis (MASH). Among those with MASH, as many as 20% may progress to cirrhosis. BAs play a significant role in both the development and management of MASH. Research into the involvement of BAs in the pathogenesis of MASH is progressively advancing. BAs are produced in the liver from cholesterol through two distinct pathways. One pathway involves the classical route, mediated by the CYP7A1 enzyme, while the other follows the acidic pathway, facilitated by the mitochondrial enzyme cytochrome P450 27α hydroxylase (CYP27A).

A wealth of research has repeatedly shown disruptions in BA metabolism in individuals with MASH. These disturbances are characterized by increased concentrations of primary conjugated BAs, a decrease in certain secondary BAs, and changes in the overall composition of BAs excreted from the body [143, 144].

There are few studies evaluating hepatic cholic acid in MASH patients. One researcher employed gas chromatography to analyze the BA composition in liver tissue samples from both individuals with MASH and healthy controls. In patients with MASH, the overall levels of BAs, along with the concentrations of cholic, chenodeoxycholic, and deoxycholic acids, were notably elevated compared to those in the control group [145]. Moreover, a strong correlation was observed between cholic acid levels and histological indicators of liver damage, such as inflammation, in patients with MASH. In contrast, higher levels of deoxycholic acid were found to be inversely related to inflammation in these patients [145]. Lake et al. [146] observed an elevation in taurine-conjugated BAs and a reduction in CA and glycocholic acid levels in patients with MASH. These findings indicate a possible shift toward an alternative pathway of BA synthesis in the context of MASH. In another study of 20 non-diabetic individuals and 22 diabetics, 77.7% of whom had MASLD, the total hepatic BA levels were significantly lower in the diabetic group compared to the control group, primarily due to a reduction in conjugated BAs [147]. In a cohort of Chinese patients, both circulating and hepatic levels of conjugated chenodeoxycholic acid were elevated in individuals with MASH. Additionally, the ratio of chenodeoxycholic acid to muricholic acid was higher in patients with MASLD compared to healthy controls. Furthermore, alterations in BA profiles in MASH were found to closely correlate with the severity of liver damage and may serve as indicators for the progression of the disease [148].

Research on BA circulation in patients with MASH has been expanding. Kalhan et al. [144] reported that fasting plasma BA levels, including glycolic acid, taurocholic acid, and taurochenodeoxycholic acid, were significantly higher in MASH patients in contrast to healthy individuals. These alterations are linked to elevated levels of taurine- and glycine-conjugated primary and secondary BAs. In patients with MASH, there was a greater variability observed in both fasting and postprandial BA profiles [149]. This suggests that the pathogenesis of liver injury, MASLD, or even MASH may be closely correlate with the elevation of BA content. Additionally, another study revealed a gradual increase in the total concentration of primary BAs, progressing from the control group to NAFL and then to MASH patients. In patients with MASH, there was an increase in total primary BAs, while total secondary BAs decreased. These changes were not linked to the presence of diabetes in individuals with NAFL [150]. Numerous studies have demonstrated that individuals with type 2 diabetes (T2DM) exhibit higher systemic concentrations of total BAs [151]. A comparison between obese individuals, those with biopsy-confirmed MASH, and healthy controls revealed that alterations in BAs were linked to the presence of insulin resistance, but not to liver necroinflammation [152]. These results underscore the intricate relationship between insulin resistance and MASLD and MASH through multiple BA metabolisms.

In conclusion, the majority of studies have indicated that patients with MASH and steatosis exhibit elevated levels of circulating total BAs, primarily due to the upregulation of conjugated BAs. However, findings regarding secondary BA levels in MASLD have been inconsistent. Some research suggests that it is the ratio of specific BAs, rather than their total concentrations or individual levels that plays a crucial role in the progression from MASLD to MASH. Additionally, insulin resistance has been identified as a key factor contributing to the dysregulation of circulating BAs. Currently, there is a limited body of research on liver BAs, and no clear consensus has been reached (Fig. 7).

**Figure 7.**
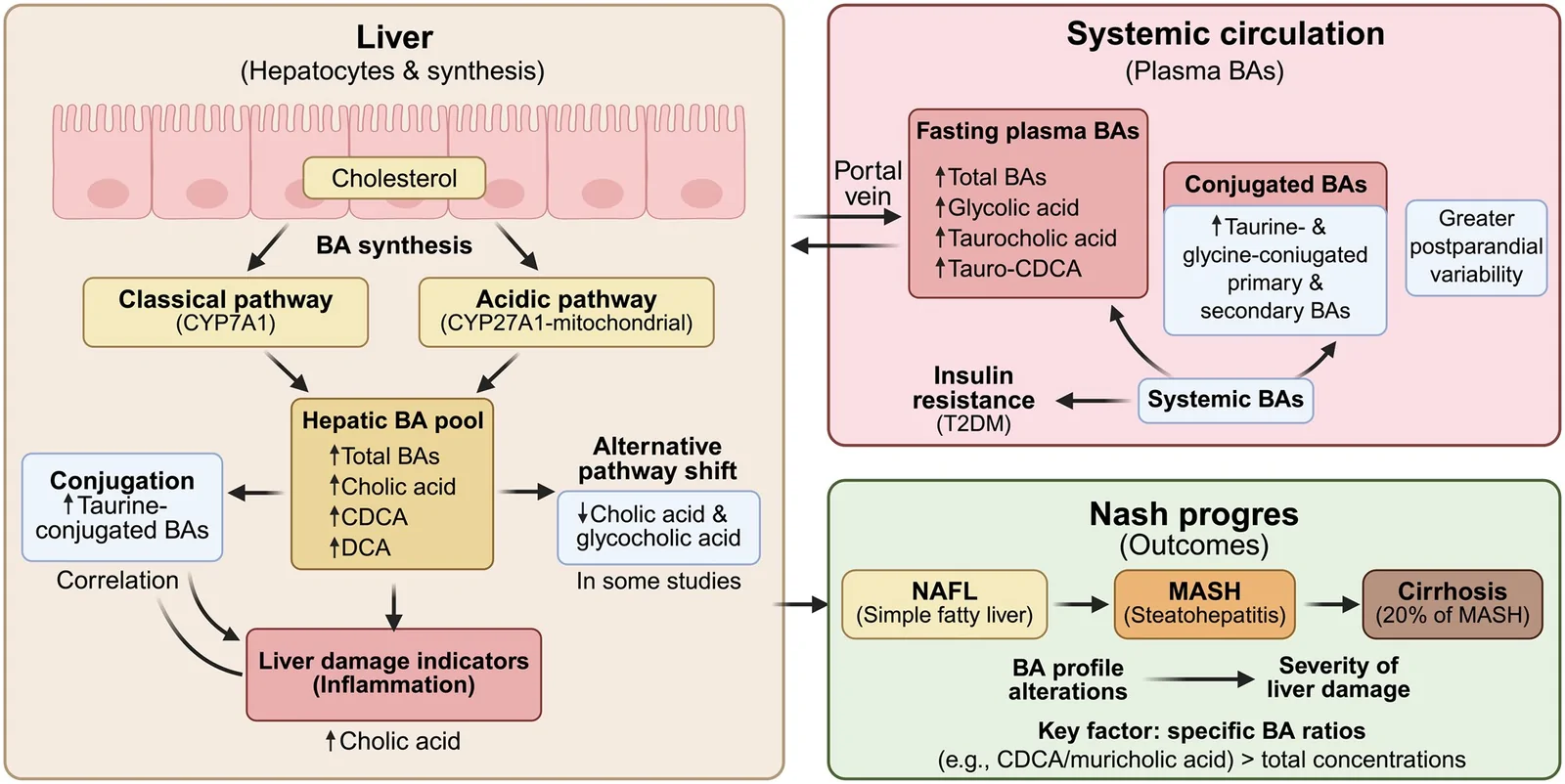
Changes in BAs in MASLD. The scheme compiles the four changes in BAs in MASLD: elevated levels of primary conjugated BAs, higher total BAs, significant association between cholic acid and histological markers of liver damage and reduced levels of specific secondary BAs. Created in BioRender. Boyu, D. (2026) BioRender.com/paf91dm.

### Hepatocellular carcinoma

HCC is the most prevalent primary liver cancer and a major cause of cancer-related mortality globally, yet there are few effective treatment options. Advanced age increases HCC vulnerability through accumulated DNA damage, cellular senescence, immune remodeling, metabolic dysfunction, chronic inflammation, and microbiome alterations. Recent studies further connect liver senescence and FBP1-regulated metabolic switching to HCC progression, RNA modifications to the transition from fatty liver to cancer, and gut microbiota to cancer initiation and therapy, underscoring that BA-driven carcinogenesis should be evaluated within an aged liver ecosystem [153].

It is well recognized that long-term elevation of circulating BAs is a potential risk factor for HCC [154]. The mechanisms through which BAs contribute to hepatocellular carcinogenesis may involve several pathways, including direct effects on hepatocytes through the generation of ROS, stimulation of cell survival and proliferation via activation of the PI3K/AKT and MAPK pathways, induction of DNA damage and apoptosis followed by compensatory proliferation, and indirect proinflammatory effects, all ultimately contributing to tumorigenesis [155]. A retrospective cohort study involving 2262 patients with CHB who underwent formal antiviral therapy found that persistently elevated serum total BAs served as an independent risk factor for HCC in these patients [156]. Another study found that a higher ratio of taurine-conjugated BAs to glycine-conjugated BAs (specifically TCDCA/GCDCA and TDCA/GDCA) was linked to an increased risk of HCC. Conversely, there was a statistically significant inverse relationship between the ratio of LCA to CDCA and the risk of HCC [157]. In a study involving diethylnitrosamine (DEN) and CA-induced tumors in mice, treatment with 0.2% CA resulted in a threefold increase in both the number and size of DEN-induced liver tumors, accompanied by a significant rise in TNF-α and IL-1β mRNA levels [158]. In a mouse model of nonalcoholic steatohepatitis–HCC (MASH–HCC) induced by streptozotocin and a high-fat diet (HFD), the liver showed increased levels of hydrophobic BAs, including DCA, TCA, TCDCA, and TLCA. Increasing the excretion of intestinal hydrophobic BAs by feeding 2% bile pigment significantly prevented HCC development in MASH–HCC model mice. The proliferation of normal human liver cell line treated with BAs was significantly increased. Additionally, in the HepG2 cell line treated with TCDCA, the expression of the tumor suppressor gene *CEBPα* was downregulated, indicating that multiple hydrophobic BAs may collectively promote liver cancer development [159].

BAs primarily signal by binding to the nuclear receptor FXR or the membrane-bound receptor TGR5, also known as G protein-coupled bile acid receptor-1 (GPBAR-1) [160]. Spontaneous hepatocarcinogenesis was observed in *FXR*^−/−^ mice, and activation of intestinal FXR could restore BAs homeostasis via the FGF15 axis to prevent liver injury from progressing to HCC [106, 161]. However, hepatocyte-specific FXR deficiency alone is not sufficient to act as a complete tumor promoter and does not cause spontaneous liver tumor development in mice. Basal BA levels in *FXR*^hep−/−^ mice were similar to those in wild-type (WT) mice, implying that liver tumorigenesis may be linked to elevated BA levels [162]. In a study involving *FXR*^−/−^*SHP*^−/−^ double knockout mice with chronically elevated BA levels, BAs were identified as upstream regulators of the Hippo pathway, promoting tumor growth by driving YAP activation. The activation of YAP by BAs was found to be concentration dependent [163]. As mentioned above, FXR has been identified as a key protective factor against carcinogenesis.

The role of the membrane-bound receptor TGR5 (GPBAR-1) in HCC appears more complex and context-dependent. Potently activated by hydrophobic BAs (LCA > DCA), TGR5 generally exerts protective, anti-inflammatory effects in the liver by inhibiting NF-κB signaling via cAMP-dependent pathways; accordingly, *Tgr5* deletion exacerbates inflammatory liver necrosis [47] (Fig. 8). However, TGR5 signaling may be subverted in established malignancies. For instance, the oncogenic α7-nAChR pathway in HCC upregulates TGR5 alongside pro-metastatic factors like MMPs and RhoA [164]. Conversely, epigenetic silencing via TGR5 promoter hypermethylation in circulating cell-free DNA (cfDNA) shows promise as a diagnostic biomarker for HCC. Given these seemingly paradoxical roles—protective against initiating inflammation yet potentially implicated in later-stage progression—elucidating the distinct temporal functions of TGR5 in hepatocarcinogenesis remains a critical frontier for future investigation.

**Figure 8.**
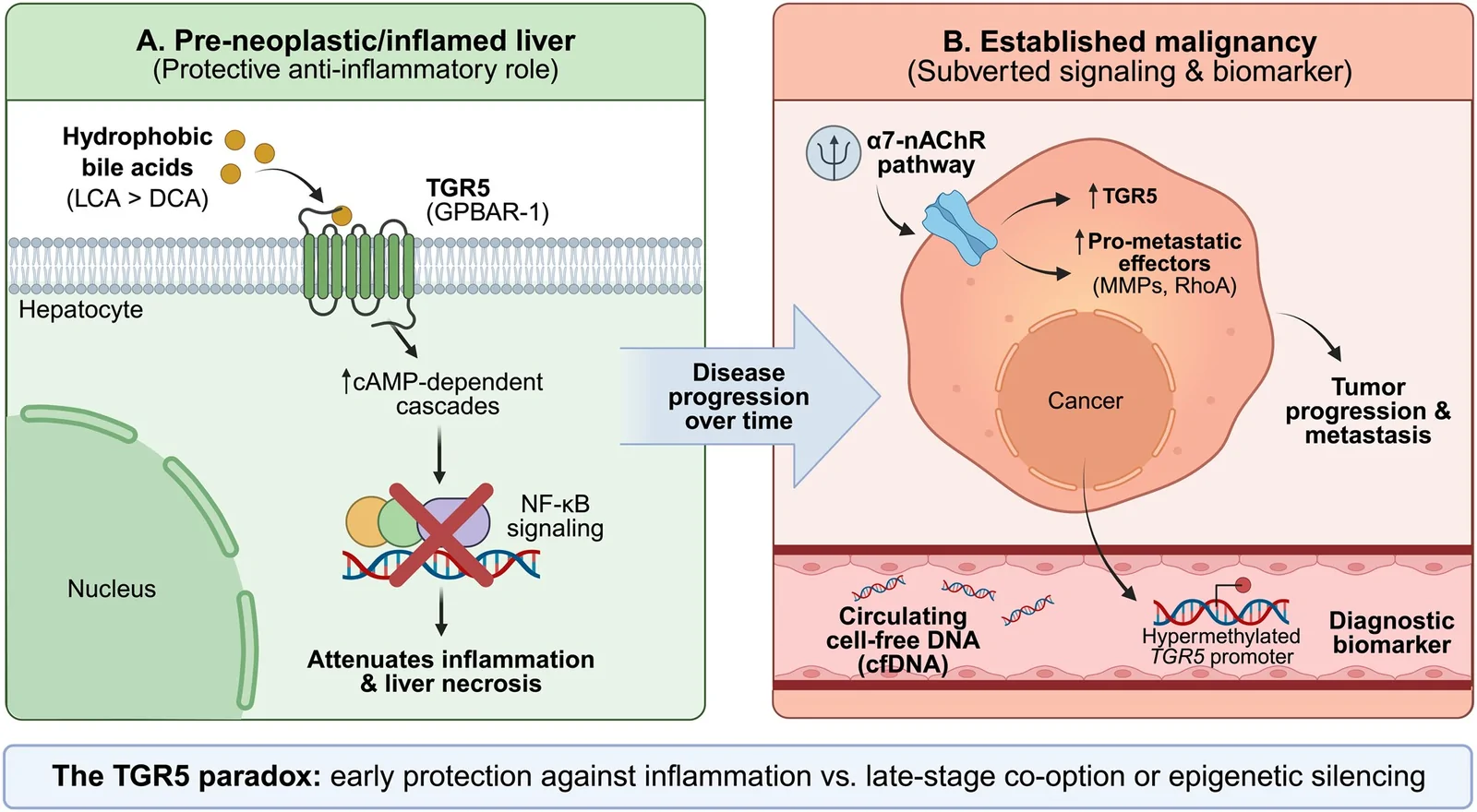
The role of TGR5 in HCC. In the pre-neoplastic or inflammatory context (left), activation of TGR5 by hydrophobic BAs (with a potency rank of LCA > DCA) exerts a hepatoprotective effect by inhibiting NF-κB signaling through cAMP-dependent pathways, thereby attenuating liver necrosis and inflammation. Conversely, in established malignancies (right), TGR5 signaling can be subverted by the oncogenic α7-nAChR pathway, leading to the upregulation of TGR5 alongside pro-metastatic factors such as MMPs and RhoA to facilitate tumor progression. Additionally, hypermethylation of the *TGR5* promoter in circulating cell-free DNA (cfDNA) acts as a potential epigenetic biomarker for HCC diagnosis, highlighting the complex temporal regulation of this receptor during hepatocarcinogenesis. Created in BioRender. Boyu, D. (2026) BioRender.com/pl858pg.

## Issues and discussion

While the reconceptualization of BAs from digestive surfactants to hormone-like signaling metabolites has transformed our understanding of hepatic metabolism, translating these insights into effective clinical therapies for aging-related liver diseases remains challenging. We emphasize three unresolved issues: the causal direction between BA remodeling and aging, the species-specific functions of individual BAs, and the need to integrate host receptors with the gut microbiome and immune system.

First, BA receptor pathways should be studied as an integrated network rather than as isolated FXR or TGR5 modules. *In vivo*, nuclear receptors and membrane receptors can exert synergistic or antagonistic effects depending on BA species, cell type, disease stage, and age. For instance, TGR5 can suppress inflammatory NF-κB signaling in macrophages [38], yet TGR5-related programs may be co-opted during established malignancy. Similarly, FXR generally protects against BA overload and carcinogenesis, but systemic FXR agonism may cause adverse effects that are clinically relevant in frail older adults. Defining the molecular switches that convert BAs from metabolic guardians to disease amplifiers in the aged microenvironment is therefore a major priority.

Second, the “one-size-fits-all” approach to BA-based diagnostics and therapeutics is limited by substantial inter-individual heterogeneity. Aging entails changes in BA synthesis, transporter expression, bile flow, gut microbial enzymatic capacity, and inflammatory tone. Because the microbiome governs the conversion of primary BAs into secondary and epimerized derivatives, age-associated dysbiosis can shift the BA pool toward hydrophobic, cytotoxic, immunomodulatory, or potentially protective species. Defining “normal” BA reference intervals therefore requires age-, sex-, diet-, and disease-stratified standards rather than a single total BA threshold.

Third, therapeutic targeting of the BA axis in older adults requires caution. FXR agonists, TGR5 agonists, BA sequestrants, UDCA/TUDCA, probiotics, and microbiota-directed approaches may all reshape BA signaling, but the same intervention may have different outcomes depending on frailty, cholestasis, renal function, polypharmacy, and microbiome composition. Restoring BA homeostasis in the aged liver may therefore require combination strategies that simultaneously reduce hydrophobic BA toxicity, restore FXR/TGR5 feedback, and remodel microbial BA metabolism.

In summary, BA dysregulation is not merely a bystander in the aging liver but a bidirectional driver and readout of aging-related hepatic vulnerability. Future research should move from reductionist receptor studies toward systems biology approaches that integrate BA species, liver cell senescence, gut microbiota, immune remodeling, and clinical aging phenotypes. Such work will be essential for developing age-specific BA-targeted strategies that improve hepatic healthspan without disrupting the fragile metabolic equilibrium of older patients.

Key unresolved questions include: (i) which BA species are causal drivers versus biomarkers of liver aging; (ii) whether protective LCA-related signals can be separated from LCA hepatotoxicity; (iii) how FXR/TGR5 agonists should be dosed in elderly patients with cholestasis or polypharmacy; (iv) how murine BA findings involving muricholic acids should be translated to humans; and (v) whether microbiota-guided BA remodeling can delay progression from MASLD/MASH, ALD, CHB, or AILDs to fibrosis and HCC.
